# Risk of lung cancer in relation to various metrics of smoking history: a case-control study in Montreal

**DOI:** 10.1186/s12885-018-5144-5

**Published:** 2018-12-19

**Authors:** T. Remen, J. Pintos, M. Abrahamowicz, J. Siemiatycki

**Affiliations:** 1 University of Montreal Hospital Research Center (CRCHUM), Montreal, Canada; 2 Department of Epidemiology and Biostatistics, McGill University, Montreal, Canada

**Keywords:** Risk assessment, Cigarette, Tobacco, Pack-years

## Abstract

**Background:**

Few epidemiologic findings are as well established as the association between smoking and lung cancer. It is therefore somewhat surprising that there is not yet a clear consensus about the exposure-response relationships between various metrics of smoking and lung cancer risk. In part this is due to heterogeneity of how exposure-response results have been presented and the relative paucity of published results using any particular metric of exposure. The purposes of this study are: to provide new data on smoking-lung cancer associations and to explore the relative impact of different dimensions of smoking history on lung cancer risk.

**Methods:**

Based on a large lung cancer case-control study (1203 cases and 1513 controls) conducted in Montreal in 1996–2000, we estimated the lifetime prevalence of smoking and odds ratios in relation to several smoking metrics, both categorical and continuous based on multivariable unconditional logistic regression.

**Results:**

Odds ratios (ORs) for ever vs never smoking were 7.82 among males and 11.76 among females. ORs increased sharply with every metric of smoking examined, more so for duration than for daily intensity. In models using continuous smoking variables, all metrics had strong effects on OR and mutual adjustment among smoking metrics did not noticeably attenuate the OR estimates, indicating that each metric carries some independent risk-related information. Among all the models tested, the one based on a smoking index that integrates several smoking dimensions, provided the best fitting model. Similar patterns were observed for the different histologic types of lung cancer.

**Conclusions:**

This study provides many estimates of exposure-response relationships between smoking and lung cancer; these can be used in future meta-analyses. Irrespective of the histologic type of lung cancer and the smoking metric examined, high levels of smoking led to high levels of risk, for both men and women.

**Electronic supplementary material:**

The online version of this article (10.1186/s12885-018-5144-5) contains supplementary material, which is available to authorized users.

## Background

The role of cigarette smoking in the etiology of lung cancer is strong and has been so recognized since at least the 1960s [1]. In the intervening years, a great deal of evidence has accumulated confirming the strong impact of smoking on cancer and extending it in various directions, such as the impact of smoking on women’s risks, the nature of dose-response relationships, the impact of quitting, the particular relationships between smoking and each of the main histologic types of lung cancer and many more [2–4]. One might imagine that all of this evidence can be put together to derive very precise estimates of the quantitative impact of smoking on lung cancer risk. But our recent review showed us that the amount of published evidence that can be assembled for meta-analyses regarding exposure-response is rather limited [5]. While there have been quite a few publications showing exposure-response results for smoking and lung cancer, they use different metrics (duration, intensity, pack-years, categorical, continuous, etc.) and different strategies for control of confounding, so that the number of results that can fairly be juxtaposed or meta-analyzed, using a common exposure metric and type of model, is limited. Providing high quality statistical evidence on smoking–lung cancer associations, including by histologic subtypes, remains an important objective because such information will be useful for public health purposes, to build lung cancer risk prediction models that can be used to advise healthy patients about their risks related to their past and possible future smoking behaviors, to understand mechanisms of carcinogenesis, and to provide information that may be useful in a legal context, for compensation or litigation purposes.

In carrying out a large case-control study of lung cancer in the Montreal area to explore the possible etiologic role of scores of occupational and environmental factors in lung cancer [6], we of course collected a detailed smoking history from each subject. We use this dataset to address two objectives: a) provide evidence of exposure-response relationships between smoking history and lung cancer, using a variety of smoking metrics and formats that might be used in future meta-analyses; b) explore the relative impact of different dimensions of smoking history and how these interact in predicting risk [7–10].

## Methods

### Cases and controls

The population-based case–control study included all lung cancer cases, males and females aged 35–75 years residing in Montreal and its surrounding suburbs and who were Canadian citizens. Histologically confirmed incident cases of lung cancer diagnosed between January 1996 and December 1997 were ascertained through active monitoring of pathology reports in the 18 participating hospitals in the metropolitan Montreal region, providing almost complete (≈98%) coverage of lung cancer diagnosis in the area. Histology of lung cancer was coded according to World Health Organization/International Agency for Research on Cancer technical report 31 [11].

Controls were randomly sampled from population-based electoral lists, frequency-matched to cases by age group (±5 years), gender and residential area. Further details about the study can be found elsewhere [12]. Ethics approval was obtained from all collaborating institutions, and written informed consent was obtained for all participants.

### Data collection

A face-to-face interview was conducted by one of our bilingual interviewers (English and French). If the subject was deceased or too ill to respond, we attempted to conduct the interview with a close next of kin proxy, usually the surviving spouse. The questionnaire was designed to collect information on sociodemographic and lifestyle characteristics, including smoking history, and a detailed semi-structured history of all jobs ever held.

#### Smoking history

Detailed self-reported information was collected about cigarette smoking habits including smoking status, ages at initiation and cessation, periods of interruption and average number of cigarettes smoked per day over the subject’s lifetime. Smokers were defined as those who smoked regularly (at least one cigarette per week) during at least 6 months, and at least 100 cigarettes in their lifetime, the others being considered “never smokers”. Since early symptoms of lung cancer can lead to changes in smoking behavior, in order to avoid reverse causality bias, we discounted the two years before index date in computing each of the smoking variables. This cutpoint of two years was recommended by Leffondré et al. [7], based on their fitting of models with different cutpoints. Thus “current smokers” were defined as subjects who still smoked at interview or had quit smoking less than 2 years before the reference date (i.e. date of diagnosis for cases and date of interview for controls), and former smokers were those who quit at least 2 years before this reference date. Smoking duration was defined as the difference between age at index date for current smokers, or age of cessation for former smokers, and age at initiation, and then subtracting total duration of any temporary cessation periods. Cumulative smoking exposure was represented by two alternative constructed variables: pack-years and cumulative smoking index (CSI). The pack-years variable was computed by multiplying the average number of cigarettes smoked per day by duration of smoking in years, and dividing by 20 (cigarettes per pack). The CSI is an index comprising all the smoking dimensions collected from study subjects in a function that is biologically motivated and that optimizes predictive power [13, 14]. Leffondre et al. proposed a modified version of CSI, adapted specifically for lung cancer, and demonstrated that the resulting aggregate exposure measure improved the fit of data, compared with conventional modeling of separate effects of different smoking components [14].

The equation is: CSI = (1–0.5^dur*/τ^) (0.5^tsc*/τ^) ln(int + 1), where

$$ {\displaystyle \begin{array}{l}\mathrm{tsc}=\mathrm{time}\ \mathrm{since}\ \mathrm{cessation},\\ {}{\mathrm{tsc}}^{\ast }=\max \left(\mathrm{tsc}-\updelta, 0\right),\\ {}\mathrm{dur}=\mathrm{duration},\\ {}{\mathrm{dur}}^{\ast }=\max \left(\mathrm{dur}+\mathrm{tsc}-\updelta, 0\right)-{\mathrm{tsc}}^{\ast },\\ {}\operatorname{int}=\mathrm{average}\ \mathrm{daily}\ \mathrm{amount}\ \mathrm{smoked}\ \mathrm{in}\ \mathrm{cigarettes},\\ {}\updelta =\mathrm{lag}\ {\mathrm{between}}^{`}{\mathrm{causalaction}}^{\prime}\mathrm{and}\ \mathrm{disease}\ \mathrm{detection},\\ {}\uptau =\mathrm{biological}\ \mathrm{half}-\mathrm{life}\ \mathrm{tabacco}\ \mathrm{of}\ \mathrm{carcinogens}\end{array}} $$

The latter two parameters, δ and τ, are estimated by trial-and-error so as to optimize the fit to data [13, 14].

#### Other covariates

Detailed information was collected on sociodemographic characteristics, including ethnicity, education and family income.

In addition, from the detailed employment history and description of each job, a team of chemists and industrial hygienists examined each completed questionnaire and translated each job into a list of potential exposures using a checklist of 294 agents that included many IARC-recognized Group 1 Lung Carcinogens [15, 16].

#### Statistical analysis

Analyses were performed separately for men and women, and either with all histologies combined or by histologic type. When simply using the term “lung cancer”, we mean all histologies combined.

All associations were estimated using multivariable unconditional logistic regression. When several variables were tested simultaneously, Wald statistics were used to compare the contribution of each variable in a model while Akaike information criterion (AIC) was used to compare the goodness of fit between the different models.

We assessed the relations between lung cancer and various smoking metrics, including duration, daily intensity, time since cessation, pack-years, and CSI. For CSI, its parameters were a priori set to values established by Leffondre et al.: half-life = 26 years and lag = 1 year (males) or 0.7 year (females) [14]. Initially the smoking metrics were analyzed one-at-a-time. Subsequently we conducted analyses with selected multiple smoking metrics in the same models. Analyses involving the time since cessation variable were performed among smokers only. For models involving all subjects, with nonsmokers being the reference group, an indicator of ever smoking was used and continuous smoking variables were centered by subtracting the mean value of the smoking variable from the original value for all smokers, while keeping 0 for never smokers [7]. For each model, the smoking variables under study and the non-smoking covariates were forced into the model.

Some analyses were conducted with continuous smoking variables transformed into categorical variables, while others were conducted on the continuous variables. For the latter, different functions were used to model the relations between continuous smoking metrics and the logit of the lung cancer risk, including (i) linear and (ii) logarithmic functions models as well as fractional polynomials (FP) [17]. In FP analyses, for each continuous variable X, one or two terms of the form X^p^ were fitted with powers p chosen from (− 2, − 1, − 0.5, 0, 0.5, 1, 2, and 3) to optimize goodness of fit, i.e. minimize the model’s deviance [17].

The following covariates were included in all models: age (continuous), respondent status (self, proxy), ethnic origin (dummy variables: French / British Isles / Italian / other Europeans / other), educational level (elementary, secondary, post-secondary), socioeconomic status (SES) as measured by median household income of the residential neighborhood, derived from census information (continuous) and exposure to those IARC Group 1 occupational lung carcinogens that had at least 1% lifetime prevalence in our study population. The following occupational exposures (lifetime prevalence as indicated) satisfied these criteria: diesel engine emissions (23.8%), crystalline silica (15.9%), benzo[a]pyrene (15.3%), chrysotile asbestos (10.9%), nickel and its compounds (6.2%), chromium VI and its compounds (4.5%) and cadmium and its compounds (2.2%). These were included in the models as qualitative ordinal variables for men: no exposure, ‘non-substantial’ exposure and ‘substantial’ exposure, where the two exposure subsets were distinguished by duration of exposure, concentration of exposure and number of hours per week of exposure. Among women, due to much lower prevalence of occupational exposures, binary variables (ever vs never exposed) were preferred.

Some sensitivity analyses were carried out with study subjects restricted to those who answered for themselves, i.e. excluding proxy responses.

The population attributable fraction (PAF) was estimated as PAF = *p*_*exp*_$\left(\frac{OR-1}{OR}\right)$ where *p*_*exp*_ represents the ratio of the number of exposed cases to the total number of cases [18]. A 95% confidence interval (CI) for the PAF was derived by replacing the point estimate of the relevant OR by, respectively, the lower and upper boundaries of the corresponding 95% CI.

All statistical analyses were performed using SAS® 9.4 software. The %MFP8 macro was used for determining the transformation of continuous variables using fractional polynomials [19].

## Results

### Selected characteristics of the study population

A total of 1434 eligible lung cancer cases were invited to participate in the study and, of those, 1203 (84%) agreed to participate. Of the 2182 population controls approached, 1513 (69%) agreed to participate. Of the 2716 participating subjects, 11 were excluded due to missing smoking information. Table 1 presents the main characteristics of the 2705 subjects included in this analysis. Briefly, 60% of the study subjects were male, a great majority were French Canadian and the mean age of our population was 63.4 years [SD = 8.5]. For both genders, cases were more likely than controls to be of French ancestry (*p* < 0.001), to have a lower educational level (*p* < 0.001) and a lower family reported income (*p* ≤ 0.001), and to have had a proxy respond on their behalf (*p* < 0.001). Adenocarcinoma was the predominant histologic type among women, whereas squamous cell carcinomas were more prevalent among men. Information about lifetime prevalence of occupational exposure to IARC Group 1 lung carcinogens of the study subjects is shown in (Additional file 1: Table S1).

**Table 1:** Selected demographic characteristics of subjects in the lung cancer study, by sex, Montreal, 1996-2000

	Men			Women		
Variables	All men (n=1,630)	Cases (n=736)	Controls (n=894)	All women (n=1,075)	Cases (n=464)	Controls (n=611)
Age (years) - Mean [SD]	64.6 ± 7.7	64.1 ± 7.9	65.0 ± 7.6	61.6 ± 9.3	61.5 ± 9.3	61.7 ± 9.3
Ethnic origin						
French	70.3%	77.5%	64.4%	72.9%	78.5%	68.7%
British Isles	5.6%	4.6%	6.4%	6.5%	9.5%	4.3%
Italian	9.4%	7.3%	11.1%	5.3%	2.8%	7.2%
Other European	8.7%	6.1%	10.8%	6.3%	4.7%	7.5%
Others	6.0%	4.5%	7.3%	9.0%	4.5%	12.3%
Education						
Elementary	39.5%	44.6%	35.4%	30.5%	36.2%	26.2%
Secondary	42.2%	42.8%	41.6%	47.4%	51.5%	44.3%
Post-secondary	18.3%	12.6%	23.0%	22.1%	12.3%	29.5%
Family reported income (k$) Mean [SD]	34.2 [14.5]	33.0 [14.9]	35.2 [14.1]	36.3 [15.4]	33.7 [15.9]	38.4 [14.7]
Respondent type						
Self	76.7%	60.2%	90.3%	82.8%	66.2%	95.4%
Proxy	23.3%	39.8%	9.7%	17.2%	33.8%	4.6%
Histologic type of lung cancer						
Adenocarcinoma		32.7%			46.6%	
Squamous cell carcinoma		35.5%			19.6%	
Small cell carcinoma		17.0%			17.2%	
Large cell carcinoma		9.6%			9.3%	
Others		5.2%			7.3%	

This table presents the demographic characteristics of the study population by sex and by case/control status

### Characteristics of smoking histories of lung cancer cases and controls

Smoking characteristics of the study population are presented in Table 2. Nearly all cases (97.6% of male cases and 93.1% of female cases) had regularly smoked cigarettes, compared to about two-thirds of controls (82.3% of men and 49.3% of women). Very few smoking cases or smoking controls had smoked for fewer than 20 years, or had averaged fewer than 20 cigarettes per day, or had accumulated fewer than 20 pack-years of smoking. As expected, compared with smoking controls, smoking cases had longer durations of smoking (*p* < 0.001), higher daily intensities (*p* < 0.001), younger ages at starting smoking (*p* < 0.001) and shorter periods since quitting smoking (*p* < 0.001).

**Table 2:** Characteristics of smoking histories of lung cancer cases and controls

			Men (N=1,630)						Women (N=1,075)					
Model	Cigarette smoking variables	Unit or category	Cases			Controls			Cases			Controls		
			N (%)		Median value	N (%)		Median value	N (%)		Median value	N (%)		Median value
a	Ever smoking	No	18	(2.5%)	-	158	(17.7%)	-	32	(6.9%)	-	309	(50.6%)	-
		Yes	718	(97.5%)	-	736	(82.3%)	-	432	(93.1%)	-	302	(49.4%)	-
Among subjects who ever smoked														
b	Age of initiation	In years: Mean [SD]	15.6 [3.4]		-	16.8 [4.0]		-	18.1 [5.4]		-	20.1 [6.9]		-
c	Smoking status	Ex-smoker	225	(31.3%)	-	478	(64.9%)	-	93	(21.5%)	-	170	(56.3%)	-
		Current smoker	493	(68.9%)	-	258	(35.1%)	-	339	(78.5%)	-	132	(43.7%)	-
d	Duration of smoking (in years)^a^	]0-20]	23	(3.2%)	15.0	141	(19.2%)	14.0	17	(3.9%)	10.0	90	(29.8%)	11.5
		]20-30]	62	(8.6%)	26.1	154	(20.9%)	26.5	65	(15.1%)	27.1	67	(22.2%)	26.0
		]30-40]	178	(24.8%)	36.0	175	(23.8%)	35.0	143	(33.1%)	35.6	80	(26.5%)	35.8
		]40-50]	267	(37.2%)	45.1	191	(25.9%)	45.3	143	(33.1%)	45.0	50	(16.5%)	44.9
		>50	188	(26.2%)	53.0	75	(10.2%)	53.4	64	(14.8%)	52.8	15	(5%)	55.0
e	Intensity of smoking (cig/day)	]0-20]	86	(12%)	20.0	250	(34%)	15.0	115	(26.6%)	15.0	170	(56.3%)	10.0
		]20-30]	327	(45.5%)	25.0	279	(37.9%)	25.0	223	(51.6%)	25.0	95	(31.4%)	25.0
		]30-40]	91	(12.7%)	40.0	68	(9.2%)	39.5	47	(10.9%)	35.0	18	(6%)	37.0
		]40-50]	144	(20.1%)	50.0	101	(13.7%)	50.0	36	(8.3%)	50.0	15	(5%)	50.0
		>50	70	(9.7%)	75.0	38	(5.2%)	75.0	11	(2.6%)	75.0	4	(1.3%)	62.5
f	Cumulative amount of smoking (in pack-years)^a^	]0-20]	24	(3.3%)	14.0	165	(22.4%)	10.0	28	(6.5%)	12.9	123	(40.7%)	7.1
		]20-40]	87	(12.1%)	33.7	169	(23%)	30.0	103	(23.8%)	32.5	95	(31.4%)	30.2
		]40-60]	204	(28.4%)	51.5	181	(24.6%)	49.4	178	(41.2%)	49.7	60	(19.9%)	49.7
		]60-80]	156	(21.7%)	65.9	111	(15.1%)	69.8	72	(16.7%)	66.3	12	(4%)	62.9
		]80-100]	64	(8.9%)	90.8	46	(6.2%)	89.7	21	(4.9%)	93.7	6	(2%)	92.5
		>100	183	(25.5%)	127.2	64	(8.7%)	125.0	30	(6.9%)	119.7	6	(2%)	116.5
g	CSI index^a^	]0-1]	33	(4.6%)	0.6	203	(27.6%)	0.6	16	(3.7%)	0.5	113	(37.4%)	0.5
		]1-2]	192	(26.7%)	1.7	309	(42%)	1.5	177	(41%)	1.7	129	(42.7%)	1.5
		]2-2.5]	288	(40.1%)	2.3	158	(21.5%)	2.2	200	(46.3%)	2.2	52	(17.2%)	2.2
		]2.5-3]	166	(23.1%)	2.7	55	(7.5%)	2.7	34	(7.9%)	2.7	8	(2.7%)	2.7
		>3	39	(5.4%)	3.2	11	(1.5%)	3.2	5	(1.2%)	3.1	0	(0%)	-
h	Time since cessation (in years)^a^	0^b^	493	(68.7%)	0	258	(35.1%)	0	339	(78.5%)	0	132	(43.7%)	0
		]0-5]	60	(8.3%)	2.7	51	(6.9%)	2.8	35	(8.1%)	2.4	35	(11.6%)	2.1
		]5-10]	50	(7%)	8.3	67	(9.1%)	7.8	20	(4.6%)	6.8	32	(10.6%)	7.8
		]10-15]	41	(5.7%)	12.6	80	(10.9%)	12.8	20	(4.6%)	12.7	19	(6.3%)	12.1
		]15-20]	41	(5.7%)	17.4	92	(12.5%)	17.9	11	(2.5%)	16.4	25	(8.3%)	18.1
		>20	33	(4.6%)	24.7	188	(25.5%)	28.0	7	(1.6%)	32.0	59	(19.5%)	24.8

This table presents the characteristics relative to cigarette smoking variables by sex among lung cancer cases and controls

^a^Computed after a discount up to 2 years when relevant

^b^i.e. current smokers or subjects who quit smoking less than 2 years before the reference date

### Lung cancer risk in relation to smoking

As expected and shown in Table 3, for both sexes, subjects who had been regular smokers were at higher risk of developing lung cancer than nonsmokers. Table 3 also shows the OR of lung cancer as a function of categories of exposure for each of several smoking metrics. Risk of lung cancer increases with duration of smoking, and with intensity of smoking, as well as with the cumulative exposure measures, Pack-years and CSI. All of the trend tests across the categories of these variables were highly statistically significant (*p* < 0.0001). For both sexes, our results highlighted a positive and quite linear association between smoking duration and the logit of the lung cancer risk. Smoking cessation at least 2 years before the reference date was associated with a reduction of the risk of lung cancer for both sexes (*p* < .0001 - data not shown). For the models exploring the role of the duration, intensity, cumulative index (i.e. pack-years) or time since cessation of smoking as categorical variables, the introduction of the second smoking variable (respectively intensity, duration, smoking status ± time since cessation, or pack-years of smoking) had no noticeable impact on the risk estimates of the primary exposure metric, though as expected, the inclusion of a second source of information on smoking in the model improved the model fit (data not shown). In both sexes, duration of smoking is a stronger predictor of risk than daily intensity of smoking, as indicated by Wald test statistics.

**Table 3:** OR estimates between various smoking-related variables (categorical) and lung cancer risk

Model	Cigarette smoking variables^a^	Unit or category	OR^b^ [95% CI]		DF	AIC
Among men						
Among all subjects (non-smokers being the reference group) - [N = 1,630 for men]						
a	Ever smoking	No	1	-	24	1907
		Yes	7.82	[4.59 - 13.30]		
b	Smoking status	Non-smoker	1	-	25	1796
		Ex-smoker	3.99	[2.31 - 6.87]		
		Current smoker	14.93	[8.66 - 25.73]		
c	Duration of smoking	0	1	-	28	1747
		]0-20]	1.23	[0.60 - 2.51]		
		]20-30]	2.98	[1.61 - 5.51]		
		]30-40]	7.84	[4.43 - 13.89]		
		]40-50]	12.82	[7.28 - 22.55]		
		>50	28.94	[15.60 - 53.66]		
d	Intensity of smoking	0	1	-	27	1848
		]0-20]	3.18	[1.78 - 5.68]		
		]20-30]	9.50	[5.50 - 16.39]		
		]30-40]	9.53	[5.11 - 17.76]		
		>40	11.87	[6.71 – 20.97]		
e	Pack-years	0	1	-	29	1762
		]0-20]	1.33	[0.67 - 2.66]		
		]20-40]	3.99	[2.21 - 7.19]		
		]40-60]	9.46	[5.38 - 16.62]		
		]60-80]	13.14	[7.30 - 23.63]		
		]80-100]	11.13	[5.74 - 21.56]		
		>100	23.64	[12.87 - 43.40]		
f	CSI index	0	1	-	27	1721
		]0-1]	1.52	[0.79 - 2.89]		
		]1-2]	5.13	[2.96 - 8.89]		
		]2-2.5]	14.80	[8.47 - 25.83]		
		>2.5	24.35	[13.38 - 44.30]		
Among subjects who ever smoked (current smokers or subjects who quit smoking less than 2 years before the reference date being the reference group) - [N = 1,461 for men]						
g	Time since cessation	0	1	-	26	1646
		]0-10]	0.45	[0.31 - 0.63]		
		]10-20]	0.27	[0.19 - 0.38]		
		>20	0.10	[0.06 - 0.17]		
Among women						
Among all subjects (non-smokers being the reference group) - [N = 1,075 for women]						
a	Ever smoking	No	1.00	-	16	1066
		Yes	11.76	[7.50 - 18.42]		
b	Smoking status	Non-smoker	1.00	-	17	1001
		Ex-smoker	4.80	[2.90 - 7.93]		
		Current smoker	21.33	[13.23 - 34.38]		
c	Duration of smoking	0	1.00	-	19	978
		]0-20]	1.51	[0.74 - 3.04]		
		]20-30]	6.37	[3.55 - 11.41]		
		]30-40]	13.64	[8.19 - 22.74]		
		>40	28.79	[16.86 - 49.16]		
d	Intensity of smoking	0	1.00	-	18	1028
		]0-20]	6.05	[3.70 - 9.90]		
		]20-30]	19.40	[11.81 - 31.86]		
		>30	18.20	[10.10 - 32.80]		
e	Pack-years	0	1.00	-	19	950
		]0-20]	2.04	[1.11 - 3.74]		
		]20-40]	8.66	[5.10 - 14.68]		
		]40-60]	25.48	[15.08 - 43.04]		
		>60	37.39	[19.79 - 70.62]		
f	CSI index	0	1.00	-	18	946
		]0-1]	1.25	[0.62 - 2.51]		
		]1-2]	11.98	[7.32 - 19.62]		
		>2	29.66	[17.67 - 49.80]		
Among subjects who ever smoked (current smokers or subjects who quit smoking less than 2 years before the reference date being the reference group) - [N = 738 for women]						
g	Time since cessation	0	1.00	-	17	799
		]0-10]	0.32	[0.20 - 0.51]		
		>10	0.15	[0.09 - 0.26]		

This table presents the association between each smoking-related (categorical) variable and the logit of the lung cancer risk by sex

^a^Computed after a discount up to 2 years when relevant

^b^Odds ratio [OR] estimated with logistic regression, adjusted for age, respondent type, education, ethnic group, annual income and exposure to lung carcinogens [each exposure being considered separately]

Models based on continuous smoking variables are presented in Table 4. For both sexes and among all the models tested, the one built with the CSI index as a linear variable provided the best fitting model, as indicated by the lowest AIC. Another model that included two smoking dimensions (i.e. pack-years and time since cessation) comes close in terms of AIC index. If for duration of smoking, a linear association with the logit of the lung cancer risk is consistently observed, the use of logarithms of the values of intensity or pack-years provided superior fit to the model that used un-transformed value. More complex fractional polynomials models, shown in footnotes of Table 4, did not substantially improve the fit over the linear or log transformation.

**Table 4:** Odds ratios between various smoking-related variables (continuous) and lung cancer risk

Model	Cigarette smoking variables^a^	Unit	β estimate	OR^b^ (95% CI)	DF	AIC	Δ AIC^c^
Among men who ever smoked [N = 1 454]							
1	Duration (Dur)	per year	(7.7 x 10^-2^) x Dur	1.08 [1.06 - 1.10]	24	1630	-
2	Intensity (Int)	per pack	0.84 x Log(Int)	2.31 [1.85 - 2.89]	24	1731	-22
3^*^	Pack-years (PY)	per pack-year	1.06 x Log(PY)	2.87 [2.35 - 3.52]	24	1646	-39
4	CSI index (CSI)	per unit	1.31 x CSI	3.70 [3.04 - 4.50]	24	1583	-
5	Duration (Dur)	per year	(7.2 x 10^-2^) x Dur	1.07 [1.06 - 1.09]	25	1602	-8
	Intensity (Int)	per pack	0.64 x Log(Int)	1.90 [1.49 - 2.42]			
6	Duration (Dur)	per year	*variable not retained in the final model*		25	1600	-10
	Intensity (Int)	per pack	0.75 x Log(Int)	2.12 [1.66 - 2.70]			
	Time since cess. (TSC)	per year	(-7.9 x 10^-2^) x TSC	0.92 [0.91 - 0.94]			
7	Pack-years (PY)	per pack-year	0.75 x Log(PY)	2.11 [1.70 - 2.61]	25	1588	-19
	Time since cess. (TSC)	per year	-0.4 x Log(TSC)	0.67 [0.60 - 0.75]			
Among women who ever smoked [N = 734]							
1^*^	Duration (Dur)	per year	(8.1 x 10^-2^) x Dur	1.08 [1.06 - 1.11]	17	781	-
2^*^	Intensity (Int)	per pack	1.22 x Log(Int)	3.40 [2.47 - 4.66]	17	792	-33
3	Pack-years (PY)	per year	1.15 x Log(PY)	3.17 [2.41 - 4.16]	17	755	-21
4	CSI index (CSI)	per unit	1.74 x CSI	5.68 [4.02 - 8.00]	17	735	-
5^*^	Duration (Dur)	per year	(6.8 x 10^-2^) x Dur	1.07 [1.04 - 1.10]	18	743	-16
	Intensity (Int)	per pack	1.05 x Log(Int)	2.87 [2.02 - 4.08]			
6^*^	Duration (Dur)	per year	(3.8 x 10^-2^) x Dur	1.04 [1.00 - 1.08]	19	739	-16
	Intensity (Int)	per pack	1.13 x Log(Int)	3.09 [2.15 - 4.45]			
	Time since cess. (TSC)	per year	(-5.0 x 10^-2^) x TSC	0.95 [0.91 - 0.99]			
7^*^	Pack-years (PY)	per pack.year	0.97 x Log(PY)	2.63 [1.97 - 3.51]	18	741	-12
	Time since cess. (TSC)	per year	-0.36 x Log(TSC)	0.70 [0.58 - 0.84]			

This table presents several model fits based on one or several smoking-related variables (continuous). Different functions were used to model the relations between continuous smoking metrics and the logit of the lung cancer risk, including linear and logarithmic functions models as well as fractional polynomials

^*^ More complex models are associated with better AIC values: the β estimates being respectively:

- for Model 3 among men: 0.78 x PY^0.5^ - 0.03 x PY - AIC = 1638

- for Model 1 among women: (3.4 x 10^-4^) x Dur3 + (-8 x 10^-5^) x Dur^3^ x log(Dur) - AIC = 773

- for Model 2 among women: -2.09 x Int^-0.5^ - AIC = 785

- for Model 5 among women: (6.6 x 10^-2^) x Dur and -1.79 x Int^-0.5^ - AIC = 740

- for Model 6 among women: (4.1 x 10^-2^) x Dur, -1.9 x Int^-0.5^ and 0.89 x TSC^-2^ - AIC = 731

- for Model 7 among women: 0.41 x PY^0.5^ and 1.03 x TSC^-2^ - AIC = 737

^a^Computed after a discount up to 2 years when relevant

^b^Odds ratio [OR] estimated with logistic regression, adjusted for age, respondent type, education, ethnic group, annual income and exposure to lung carcinogens [each exposure being considered separately – cf. the method section]

^c^Difference in AIC criterion with linear model

### Histological types

Table 5 shows ORs for selected smoking metrics with men and women combined to optimize power, for all lung cancers combined and for each histologic type. All histologic types of lung cancer were strongly associated with smoking. The ORs between ever regular smoking and lung cancer were close to 10, considering the statistical variability, for each of squamous cell, large cell and adenocarcinoma, and it was undefined for small cell lung cancer because there were no non-smokers among the 205 cases.

**Table 5:** OR estimates between various smoking-related variables (categorical) and lung cancer risk by histology, males and females combined

				All histologies combined			Squamous cell carcinoma			Small cell carcinoma			Adenocarcinoma			Large cell carcinoma		
Model	Cigarette smoking variables^a^	Category	N (controls)	N (cases)	OR^b^ [95% CI]		N (cases)	OR^b^ [95% CI]		N (cases)	OR^b^ [95% CI]		N (cases)	OR^b^ [95% CI]		N (cases)	OR^b^ [95% CI]	
Among all subjects (non-smokers being the reference group*)*																		
a	Ever smoking	No	467	50	1	-	12	1	-	0	1	-	26	1	-	4	1	-
		Yes	1038	1150	9.56	[6.88 - 13.30]	340	9.44	[5.14 - 17.35]	205	Undefined		431	8.48	[5.46 - 13.17]	110	11.27	[3.94 - 32.18]
b	Smoking status	Non-smoker	467	50	1	-	12	1	-	0	1	-	26	1	-	4	1	-
		Ex-smoker	648	318	4.47	[3.15 - 6.34]	115	4.99	[2.65 - 9.40]	39	Undefined		122	4.02	[2.51 - 6.44]	22	3.86	[1.25 - 11.88]
		Current smoker	390	832	17.59	[12.47 - 24.79]	225	17.38	[9.31 - 32.44]	166			309	15.16	[9.62 - 23.89]	88	24.10	[8.25 - 70.37]
c	Duration of smoking	0	467	50	1	-	12	1	-	0	1	-	26	1	-	4	1	-
		]0-30]	452	167	3.07	[2.11 - 4.47]	32	2.20	[1.09 - 4.44]	28	Undefined		76	3.00	[1.82 - 4.94]	19	3.81	[1.21 - 12.01]
		]30-40]	255	321	10.91	[7.57 - 15.74]	93	10.67	[5.58 - 20.39]	57			121	9.97	[6.10 - 16.27]	32	15.14	[4.92 - 46.58]
		>40	331	662	22.20	[15.42 - 31.96]	215	21.23	[11.21 - 40.20]	120			234	21.25	[12.99 - 34.75]	59	33.24	[10.52 - 105.05]
d	Intensity of smoking	0	467	50	1	-	12	1	-	0	1	-	26	1	-	4	1	-
		]0-20]	420	201	4.79	[3.34 - 6.87]	43	3.59	[1.83 - 7.05]	39	Undefined		87	4.46	[2.75 - 7.22]	20	5.87	[1.90 - 18.10]
		]20-30]	374	550	14.12	[9.93 - 20.08]	148	13.80	[7.32 - 26.00]	90			231	14.26	[8.92 - 22.79]	54	18.03	[6.04 - 53.75]
		>30	244	399	15.31	[10.53 - 22.26]	149	17.59	[9.19 - 33.65]	76			113	11.22	[6.75 - 18.65]	36	17.20	[5.46 - 54.10]
e	Pack-years	0	467	50	1	-	12	1	-	0	1	-	26	1	-	4	1	-
		]0-30]	423	119	2.94	[2.01 - 4.31]	28	2.52	[1.24 - 5.11]	19	Undefined		55	2.85	[1.71 - 4.76]	9	2.56	[0.74 - 8.84]
		]30-60]	370	505	13.60	[9.55 - 19.35]	118	11.08	[5.85 - 20.93]	82			229	14.50	[9.06 - 23.19]	50	19.28	[6.36 - 58.37]
		>60	245	526	24.16	[16.57 - 35.41]	194	26.40	[13.76 - 50.63]	104			147	17.96	[10.75 - 30.01]	51	40.61	[12.58 - 131.09]
f	CSI index	0	467	50	1	-	12	1	-	0	1	-	26	1	-	4	1	-
		]0-1]	316	49	1.64	[1.05 - 2.56]	11	1.20	[0.51 - 2.82]	5	Undefined		22	1.61	[0.87 - 2.96]	4	1.54	[0.36 - 6.51]
		]1-2]	438	369	8.19	[5.76 - 11.64]	101	7.96	[4.20 - 15.07]	59			155	7.79	[4.86 - 12.47]	36	10.87	[3.58 - 33.01]
		>2	284	732	24.68	[17.22 - 35.37]	228	23.90	[12.68 - 45.04]	141			254	22.50	[13.91 - 36.39]	70	37.04	[12.03 - 114.04]

This table presents the association between each smoking-related (categorical) variable and the logit of the lung cancer risk by histology

^a^Computed after a discount up to 2 years when relevant

^b^Odds ratio [OR] adjusted for age, sex, respondent type, education, ethnic group, annual income and exposure to lung carcinogens [each exposure being considered separately

### Sensitivity analysis limited to self-respondents

Since cases were more likely than controls to have had a proxy respond on their behalf, we adjusted for proxy status in the main analyses. But, as a further insight into the possible impact of using proxies, we re-ran some analyses among self-respondents only, and compared these with the results of combining self and proxy respondents (see Additional file 2: Table S2). Among men, the adjusted OR for a binary indicator of ever smoking and lung cancer was 7.82 (95% CI [4.59–13.30]) in analyses that included all respondents, and 6.96 (95% CI [3.82–12.68]) in analyses restricted to self-respondents. Among women the OR was 11.76 (95% CI [7.50–18.42]) in analyses including all respondents, and 12.17 (95% CI [7.45–19.85]) in analyses restricted to self-respondents. When considering the CSI index, the OR corresponding to a one unit increase in CSI was slightly lower among self-respondents than among all respondents. But in all of these contrasts, the confidence limits between self-respondent and all respondent results overlapped considerably, and none of the substantive inferences would have changed.

### Population attributable fraction

Given the overall OR estimate, its confidence limits and the observed prevalence of smoking among cases (96%), we estimate that the Population attributable fraction was 0.858 (0.819–0.887). The estimates were almost identical in men and women.

## Discussion

Although tobacco smoking is the main cause of lung cancer in humans, there is no widely accepted estimate of the exposure-response relationship between smoking and lung cancer. This is partly due to a false impression that the smoking-lung cancer association is so well established that there is little to be learned from additional attention to the issue. Because the analysis and description of dose-response relationships involves such idiosyncratic methodologies and parametrizations, there are really not a tremendous number of published results that can be usefully assembled for meta-analyses or other attempts at synthesizing knowledge. Finally, there remains a great deal to learn about the mechanisms of carcinogenesis by studying valid and generalizable dose-response relationships. The addition of new evidence from studies such as ours will hopefully increase the likelihood that reasonably representative estimates can be derived from the world body of evidence.

As well as describing the smoking history of a North American study population at the end of the twentieth century, this paper provides new estimates of the relationship between cigarette smoking and lung cancer risk for both sexes.

There were significant changes in design of cigarettes (filters, “light”, etc.) in the 1960s and 1970s, and in contrast with many previous studies that had been conducted from the 1950s to the 1980s and that evaluated risks of smoking the types of cigarettes marketed in the middle of the twentieth century, our study covers a population that was largely exposed to the types of cigarettes that came into widespread usage in the last part of the twentieth century. Cigarette smoking was a prevalent habit in this population, certainly among men, but even among women. Among men, 97.5% of cases and 82.3% of controls had ever smoked regularly, while among women, it was 93.1 and 49.4%. These numbers are in the same order of magnitude as those observed in a previous case-control study conducted in Montreal in the 1980’s [20] and in others in the United States [21]. Further, there were very few short duration or low daily intensity smokers.

To provide useful and relevant information for various purposes, different parametrizations and different statistical risk models were created. There are many more published results with categorical parametrization of smoking variables, namely duration, intensity and pack-years, than there are with any particular continuous parametrizations of smoking variables. There are more ways to model the shapes of continuous variables, while categorizations have more limited options for summarization. While there are advantages to the flexibility of continuous variable modelling in a given study, when a meta-analysis of many studies is called for, using the more common categorical variables is a useful strategy. Models were also built either with each smoking variable studied separately or combined with each other.

For the association between binary smoker/nonsmoker variable and lung cancer, we observed an OR of 7.82 [4.59–13.30] for men, a risk slightly lower than the one we derived from a meta-analysis covering studies conducted in North America and Europe [5]. Our estimate is slightly higher than the one (OR = 6.18 95% CI [5.49–6.95]) observed in a recent meta-analysis based on a larger number of studies [3], but those included studies in other parts of the world which were not comparable to ours in terms of history of smoking and ethnic profile of the population. For women, the corresponding OR is 11.76 [7.50–18.42], which is higher than the one (OR = 4.43 95% CI [3.84–5.10]) computed in the meta-analysis cited above [3]. Gender susceptibility to cigarette smoking-attributable lung cancer, a topic under debate in recent years [22–26], will be addressed in a separate paper.

There is more published evidence concerning smoking duration and intensity as distinct predictors of risk than any other metrics. Of these, duration of regular smoking seems to be the more important predictor of lung cancer risk [4]. For both sexes, our results highlighted a positive and quite linear association between smoking duration and the logit of the lung cancer risk. In our analyses, particular attention was paid to the reconstruction of the smoking duration variable which excluded any temporary or permanent periods of cessation. It is conceivable that the lower predictive value of smoking intensity is due to the fact that duration of smoking may be recalled and reported with greater accuracy than average daily intensity over the lifetime smoking history. Obtaining a good estimate of smoking intensity is very challenging due to the potential variability in the true number of cigarettes smoked per day over different periods within a long smoking “career”, and the difficulty of recalling such variability over a long time span. The estimate used in our study was based on the reported average number of cigarettes smoked per day throughout the smoking history of the subject, an estimate often used in epidemiological studies [27]. Log-transformation for intensity of smoking yielded best fit of the data, suggesting that the impact of increasing daily intensity by a fixed number of cigarettes per day is more harmful for light smokers than for heavy smokers, as suggested elsewhere [10, 28].

“Pack-years” smoked, calculated as the average number of packs of cigarettes smoked per day, multiplied by the cumulative number of years during which a person smoked, is the simplest and most commonly reported cumulative smoking exposure metric. Use of the “pack-year” index has been criticized [8, 29, 30] on the grounds that it gives equivalent weight to intensity and duration as contributors to the risk of lung cancer, and it does not explicitly account for time since quitting. Nevertheless, the pack-years variable has the virtue of simplicity, it partially accounts for time since quitting since length of time since quitting implies shorter duration and is a strong predictor of the risk of various smoking-related diseases [31–33]. For some purposes, this simple but powerful metric is perfectly adequate, while for other purposes, more sophisticated treatment of the components of a smoking history might be indicated. The CSI [34] is one such possible metric and as shown by Leffondre et al [14] and here, it is a very effective smoking metric, and indeed it performed very well compared with other models in predicting lung cancer risk.

In models using continuous smoking variables, all metrics had strong effects on OR and mutual adjustment among smoking metrics did not noticeably attenuate the OR estimates, indicating that each metric carries some independent risk-related information.

In any case, each of these metrics is an imperfect measure of inhaled dose because of intra and inter-individual variation in: (i) depth of inhalation, (ii) number of puffs taken per cigarette and (iii) retention time in the lung [4].

Finally, in our study population, we estimated that, irrespective of gender, about 86% (95% CI [81–89%]) of the lung cancer cases were attributable to cigarette smoking. That proportion is consistent with previous findings [35, 36].

## Conclusion

Besides presenting a portrait of the smoking-lung cancer association in North America at the end of the twentieth century, this study provides a panorama of estimates of this association derived from several modeling strategies and several parameterizations of the smoking variables. This provides new material for future meta-analyses using any of the smoking metrics presented here. Among the notable substantive findings are: the high risk estimates of all types of lung cancer due to smoking despite the changes over time in composition and tar output of cigarettes; the high risk estimates among women, and consequent high attributable fractions; the clear message of increased risk with even the simplest metrics of exposure; and the apparently stronger influence of duration of smoking than daily amount smoked on risk of lung cancer.

## Additional file

Additional file 1: **Table S1.** Lifetime prevalence of occupational exposure to IARC Group 1 lung carcinogens of the study subjects. This table presents the lifetime prevalence of occupational exposure to IARC Group 1 lung carcinogens (diesel engine emissions, crystalline silica, benzo[a]pyrene, chrysotile asbestos, nickel and its compounds, chromium (VI), cadmium and its compounds, arsenic and its compounds, beryllium and its compounds) by category of exposure (non-exposed, not substantial exposure or substantial exposure). (DOCX 18 kb)

Additional file 2: **Table S2.** OR estimates between smoking-related variables and lung cancer risk by respondent type. This table presents the association between ever smoking (or CSI) and the logit of the lung cancer risk by respondent type. (DOCX 15 kb)

## Abbreviations

AIC: Akaike information criterion
CSI: Cumulative Smoking Index
FP: Fractional polynomial
IARC: International Agency for Research on Cancer
PAF: Population attributable fraction
SES: Socioeconomic status
