# Supplementary material for: Risk of lung cancer in relation to various metrics of smoking history: a case-control study in Montreal
Source: BMC Cancer. 2018 Dec 19;18:1275. doi: 10.1186/s12885-018-5144-5 (PMC6299933; doi:10.1186/s12885-018-5144-5)
Supplement: Supplementary file 1 — Table S1. Lifetime prevalence of occupational exposure to IARC Group 1 lung carcinogens of the study subjects. This table presents the lifetime prevalence of occupational exposure to IARC Group 1 lung carcinogens (diesel engine emissions, crystalline silica, benzo[a]pyrene, chrysotile asbestos, nickel and its compounds, chromium (VI), cadmium and its compounds, arsenic and its compounds, beryllium and its compounds) by category of exposure (non-exposed, not substantial exposure or substantial exposure). (DOCX 18 kb) [file 12885_2018_5144_MOESM1_ESM.docx]

# Table S1: Lifetime prevalence of occupational exposure to IARC Group 1 lung carcinogens of the study subjects

|  | Both sex | Men | | | Women | | |
| --- | --- | --- | --- | --- | --- | --- | --- |
| Variables | All subjects (n=2705) | All men  (n=1630) | Cases (n=736) | Controls (n=894) | All women (n=1075) | Cases (n=464) | Controls (n=611) |
| Diesel engine emissions |  |  |  |  |  |  |  |
| - Non-exposed | 76.1% | 62.3% | 59.1% | 64.9% | 97.2% | 97.2% | 97.2% |
| - Not substantial exposure | 16.9% | 26.3% | 26.8% | 25.9% | 2.5% | 2.8% | 2.3% |
| - Substantial exposure | 7.0% | 11.4% | 14.1% | 9.2% | 0.3% | 0% | 0.5% |
| Crystalline silica |  |  |  |  |  |  |  |
| - Non-exposed | 84.0% | 78.0% | 75.7% | 80.0% | 93.1% | 92.0% | 93.9% |
| - Not substantial exposure | 12.8% | 16.8% | 17.9% | 15.9% | 6.7% | 7.8% | 5.9% |
| - Substantial exposure | 3.2% | 5.1% | 6.4% | 4.1% | 0.2% | 0.2% | 0.2% |
| Benzo[a]pyrene |  |  |  |  |  |  |  |
| - Non-exposed | 84.6% | 75.1% | 72.3% | 77.4% | 99.1% | 99.4% | 98.8% |
| - Not substantial exposure | 12.7% | 20.5% | 21.5% | 19.8% | 0.8% | 0.6% | 1.0% |
| - Substantial exposure | 2.7% | 4.4% | 6.2% | 2.8% | 0.1% | 0% | 0.2% |
| Chrysotile asbestos |  |  |  |  |  |  |  |
| - Non-exposed | 89.0% | 82.1% | 81.1% | 82.9% | 99.5% | 100% | 99.2% |
| - Not substantial exposure | 8.5% | 13.7% | 14.8% | 12.9% | 0.5% | 0% | 0.8% |
| - Substantial exposure | 2.5% | 4.2% | 4.1% | 4.2% | 0% | 0% | 0% |
| Nickel and its compounds |  |  |  |  |  |  |  |
| - Non-exposed | 93.7% | 90.1% | 89.9% | 90.3% | 99.3% | 99.4% | 99.2% |
| - Not substantial exposure | 4.6% | 7.3% | 7.6% | 6.9% | 0.6% | 0.6% | 0.6% |
| - Substantial exposure | 1.6% | 2.6% | 2.5% | 2.8% | 0.1% | 0% | 0.2% |
| Chromium (VI) |  |  |  |  |  |  |  |
| - Non-exposed | 95.5% | 93.2% | 93.6% | 92.8% | 99.1% | 99.1% | 99.0% |
| - Not substantial exposure | 3.4% | 5.1% | 4.4% | 5.7% | 0.8% | 0.9% | 0.8% |
| - Substantial exposure | 1.1% | 1.7% | 2.0% | 1.5% | 0.1% | 0% | 0.2% |
| Cadmium and its compounds |  |  |  |  |  |  |  |
| - Non-exposed | 97.8% | 96.6% | 96.2% | 96.9% | 99.6% | 99.6% | 99.7% |
| - Not substantial exposure | 1.8% | 2.7% | 2.8% | 2.6% | 0.4% | 0.4% | 0.3% |
| - Substantial exposure | 0.4% | 0.7% | 1.0% | 0.5% | 0% | 0% | 0% |
| Arsenic and its compounds |  |  |  |  |  |  |  |
| - Non-exposed | 99.1% | 98.7% | 99.5% | 98.0% | 99.9% | 100% | 99.8% |
| - Not substantial exposure | 0.6% | 0.9% | 0.4% | 1.3% | 0.1% | 0% | 0.2% |
| - Substantial exposure | 0.3% | 0.4% | 0.1% | 0.7% | 0% | 0% | 0% |
| Beryllium and its compounds |  |  |  |  |  |  |  |
| - Non-exposed | >99.9% | 99.9% | 99.9% | 100% | 100% | 100% | 100% |
| - Not substantial exposure | <0.1% | 0.1% | 0.1% | 0% | 0% | 0% | 0% |
| - Substantial exposure | 0% | 0% | 0% | 0% | 0% | 0% | 0% |
