# Supplementary material for: Risk of lung cancer in relation to various metrics of smoking history: a case-control study in Montreal
Source: BMC Cancer. 2018 Dec 19;18:1275. doi: 10.1186/s12885-018-5144-5 (PMC6299933; doi:10.1186/s12885-018-5144-5)
Supplement: Supplementary file 2 — Table S2. OR estimates between smoking-related variables and lung cancer risk by respondent type. This table presents the association between ever smoking (or CSI) and the logit of the lung cancer risk by respondent type. (DOCX 15 kb) [file 12885_2018_5144_MOESM2_ESM.docx]

Table S2: OR estimates between smoking-related variables
and lung cancer risk by respondent type

| Cigarette smoking variables | All respondents | | | Self-respondents | | |
| --- | --- | --- | --- | --- | --- | --- |
|  | N ^a^ | OR^b^ [95% CI] | | N ^a^ | OR^b^ [95% CI] | |
| Among men | | | | | | |
| Ever smoking (Yes/No) | 1630 | 7.82 | [4.59 - 13.30] | 1250 | 6.96 | [3.82 - 12.68] |
| per unit of CSI  (restricted to smokers) | 1454 | 3.70 | [3.04 - 4.50] | 1092 | 3.49 | [2.81 - 4.34] |
| Among women | | | | | | |
| Ever smoking (Yes/No) | 1075 | 11.76 | [7.50 - 18.42] | 890 | 12.17 | [7.45 - 19.85] |
| per unit of CSI  (restricted to smokers) | 734 | 5.75 | [4.08 - 8.11] | 573 | 5.02 | [3.52 - 7.17] |

^a^ N= total number of subjects (cases plus controls) in the analysis.

^b^ Odds ratio [OR] adjusted for age, sex, respondent type, education, ethnic group, annual income and exposure to lung carcinogens [each exposure being considered separately] / some occupational exposure variables were removed for the model based on proxy respondent to avoid instability in model
